# Growth Inhibition of *Streptococcus* from the Oral Cavity by α-Amyrin Esters

**DOI:** 10.3390/molecules171112603

**Published:** 2012-10-25

**Authors:** Gloria Díaz-Ruiz, Liliana Hernández-Vázquez, Héctor Luna, María del Carmen Wacher-Rodarte, Arturo Navarro-Ocaña

**Affiliations:** 1 Departamento de Alimentos y Biotecnología, Facultad de Química, UNAM. Circuito Exterior, Ciudad Universitaria 04510, Mexico; Email: gloria_druiz@yahoo.com.mx (G.D.-R.); wacher@unam.mx (M.C.W.-R.); 2 Departamento de Sistemas Biológicos, Universidad Autónoma Metropolitana-Xochimilco, Calz. del Hueso 1100, Col. Villa Quietud 04960, Mexico; Email: lhernandez@correo.xoc.uam.mx (L.H.-V.); lchm1964@correo.xoc.uam.mx (H.L.)

**Keywords:** cariogenic microorganism, amyrin esters, antimicrobial activity

## Abstract

Five terpenoids were tested by the macrodilution broth method to determine their inhibition activity on cariogenic bacterial growth. In general, α-, β-amyrin and α-amyrin phenylacetate proved to be active, reducing the bacterial viability to less than 20%.

## 1. Introduction

**Figure 1:**
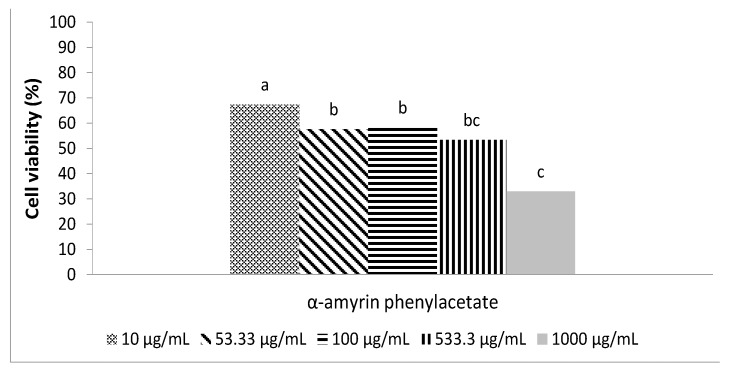
Viability of *Streptococcus mutans* after 24 h exposure to α-amyrin phenylacetate at the concentrations used in this study. The viability was adjusted using BHI broth + Tween 80 as a control (100%).The initial cellular concentration was adjusted to 10^3^ cfu/mL. ^abc^ indicates if there is a statistically significant difference between concentrations by derivate (Kruskal-Wallis test, α = 0.05). A different letter indicates there is a statistically significant difference.

The terpenoids α-amyrin (**3**) and β-amyrin (**5**) are commonly found in medicinal plants and oleo-resins obtained by bark incision of several species of *Bursera* or *Protium spp.* of the Burseraceae family (Figure 1) [1]. They have shown various pharmacological activities *in vitro* and *in vivo* against several health-related conditions, including microbial, fungal, and viral infections, cancer and inflammation. In addition, there are abundant examples of α-amyrin-containing plants used in folk medicine. For example *Strobilanthes callosus*, which has anti-inflammatory and antimicrobial activities [2], and *Decalepis hamiltonii*, which is used as an appetite stimulant, blood purifier and food preservative [3,4].

α/β-Amyrin modulate acute periodontal inflammation by reducing neutrophils infiltration, oxidative stress, and the production of pro-inflammatory cytokine TNF-α, which suggests they might be useful as therapeutic agents for the treatment of gingivitis and periodontitis [5]. Other studies have demonstrated that the α/β-amyrin mixture also has anti-inflammatory, gastroprotective, antiallergenic and antinociceptive activities [6].

The antifungal activity of amyrin and some 3-*O*-acyl derivatives has been evaluated against *Candida* species. Additionally, inhibition of the adhesion of *C. albicans* to human epithelial cells *in vitro* was also determined. Amyrin formate and acetate derivatives were the most active compounds, inhibiting all the opportunistic *Candida* species when tested in concentrations from 30 to 250 mg/mL. The formate also showed interesting inhibition of the adhesion ability of *C. albicans* [7]. β-Amyrin acetate isolated from *Heliotropium marifolum* showed potent activity against *Penicillum chrysogenum*, *Escherichia coli* and *Klebsiella pneumonia* [8].

Dental caries is a common bacterial pathology caused by a biofilm consisting of a multitude of microorganisms present on the tooth surface [9], being streptococci the bacteria most frequently associated with cariogenesis [10]. One method for caries prevention is the use of antimicrobial agents to limit the growth of microorganisms such as *Streptococcus mutans*. Extracts of miswak, tea tree oil, peppermint, green tea and manuka honey have all been recently incorporated into mouth rinses and toothpastes [11,12,13,14].

Some compounds with potential activity against oral microorganisms have been found in Nature, e.g., *S. mutans* was sensitive to totarol, a diterpenoid isolated from the bark of *Podocarpus nagi* with MICs of 0.78 μg/mL and MBC of 0.78 μg/mL [15]. The methanolic extract from the bark of *B. crassifolia* and the β-amyrin isolated therefrom inhibited the growth of bacteria, including *S. mutans*, at concentrations ranging from 64 to 1088 μg/mL; the minimum inhibitory concentration (MIC) for β-amyrin was 128 μg/mL [16].

The aim of this work was to isolate β-amyrin from nanche (*Byrsonima crassifolia*) and α-amyrin from copal (*Bursera* spp) and prepare some esters for the evaluation of their capacity to inhibit the growth of oral streptococci associated with dental caries.

## 2. Results and Discussion

### 2.1. Isolation of α- and β-Amyrin

The triterpenes α-amyrin (**3**) and β-amyrin (**5**) were isolated from copal and nanche bark in 7.0% and 2.0%, respectively [17]. Because in preliminary studies a greater activity was observed with α-amyrin than with β-amyrin, we decided to prepare some esters of α-amyrin to assay their activity against cariogenic microorganisms.

### 2.2. Esterification of α-Amyrin

**Scheme 1:**
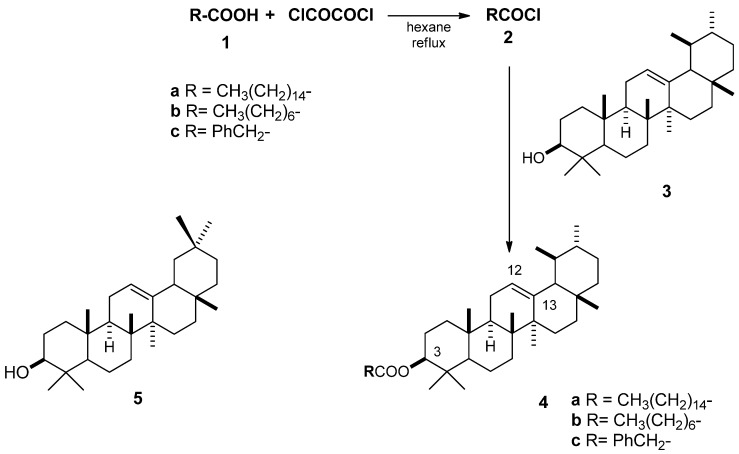
Structure of α and β-amyrin and esterification reactions of α-amyrin.

Three esters of α-amyrin **4a**–**c** were synthesized in good to excellent yields (70–98%) (Scheme 1). The spectroscopic data of **4a** and **4b** are in agreement with those reported in the literature [6,18]. To the best of our knowledge, α-amyrin phenylacetate (**4c**) is reported and completely characterized for the first time. Its IR spectra showed an absorption band at 1724 cm^−1^ assigned to C=O of the ester group, and absorption bands between 1404–1583 of (C=C) for the aromatic ring. In the ^1^H-NMR spectrum of **4c**, signals at 0.75–2.03 ppm showed the characteristic terpene profile. The protons alpha to the C=O group of the phenylacetate fragment appeared as a singlet at 3.72 ppm; H-3 appeared as a double-doublet at 4.48 ppm with a *J* = 9.2, 5.6 Hz; and H-12 as a triplet at 5.11 ppm, with a *J* = 3.6 Hz. The five protons corresponding to the aromatic fragment appeared as multiplets at 7.24–7.33 ppm. In the ^13^C-NMR the signal for C-5 was at 55.0 ppm, C-3 at 81.2 ppm, C-12 at 123.9 ppm, C-13 at 139.3 ppm, and the C=O appeared at 170.9 ppm.

### 2.3. Antimicrobial Activity Using the Macrodilution Broth Method

In this work, five compounds (namely **3**, **4a**–**c**, **5**) were screened for antimicrobial activity using the macrodilution broth method, which is commonly use in the susceptibility assay for the antimicrobial evaluation of organic compounds [19]. Two concentrations of *S. mutans* were used, a lower (1 × 10^3^ cfu/mL) and a higher concentration (1 × 10^6^ cfu/mL), because a low concentration of *S. mutans* is associated with caries-free subjects, and the tooth surfaces susceptible to becoming carious are associated with a higher concentration [20].

During the experiments, chlorhexidine dihydrochloride (CHX) was used as a positive control, since produced 0% cell viability for all the assayed strains. The negative control consisted of a broth solution with 3.6 µL of Tween 80, which resulted in 100% cell viability for all strains tested.

To obtain information about the dosis-response relationship, *S. mutans* (at 10^6^ cfu/mL) was exposed to the five compounds α-amyrin (**3**), β-amyrin (**5**), α-amyrin palmitate (**4a**), α-amyrin octanoate (**4b**) and α-amyrin phenylacetate (**4c**) (Scheme 1), at the following concentrations: 10, 53.33, 100, 533.33, 1,000 μg/mL. The results shown in Table 1 indicate that the palmitate (83–43% viability) and the octanoate esters were less active (89–39% viability), the phenylacetate (19–9% viability) being the most active, followed by α-amyrin (**3**) and β-amyrin (**5**). It is worth mentioning that the effect of the highest concentration of α-amyrin (16% viability) was comparable with the effect produced by the lowest concentration of α-amyrin phenylacetate (19% viability), which proved to be about 100 times more active than α-amyrin (**3**). The results showed statistically significant differences between concentrations according the Kruskal-Wallis test, at α = 0.05.

**Table 1:** Percentage of viability of *Streptococcus mutans* after 24 h exposure to the five terpenes at the concentrations used in this study. The viability was adjusted using BHI broth + Tween 80 as a control (100%).The initial cellular concentration was adjusted to 10^6^ cfu/mL.

Compound	Terpene concentration (μg/mL)				
	10	53.33	100	533.33	1000
α-amyrin (**3**)	62	46	25	25	16
β-amyrin (**5**)	72	61	58	46	37
α-amyrin palmitate (**4a**)	83	82	68	46	43
α-amyrin octanoate (**4b**)	89	62	60	40	39
α-amyrin phenylacetate (**4c**)	19	16	12	12	9

Continuing the study, activity against *S. salivarius*, *S. sanguinis*, *S. oralis* (at 1 × 10^6^ cfu/mL) was tested, but only with the three most active compounds, using the same range of concentrations, and the results are shown in Table 2.

**Table 2:** Percentage of viability, related to terpene concentration, of *Streptococcus salivarius*, *Streptococcus sanguinis and Streptococcus oralis* after 24 h exposure to three selected derivatives at the concentrations used in this study 10–1,000 (µg/mL). The viability has been adjusted using BHI broth + Tween 80 as a control (100%).The initial cellular concentration was adjusted to 10^6^ cfu/mL.

Compound	*S. salivarius*	*S. sanguinis*	*S. oralis*
α-amyrin (**3**)	68–56%	56–20%	95–47%
β-amyrin (**5**)	68–57%	90–63%	100–46%
α-amyrin phenylacetate (**4c**)	86–12%	83–48%	3–1%

The range of cell development was related to the concentration of the three terpenes used for the experiments. In general, α-amyrin phenylacetate (**4c**) was the most active, especially at higher concentrations, except against *S. sanguinis*, for which α-amyrin was slightly more active at all the concentrations tested. For *S. oralis*, the inhibition activity of **4c** was outstanding at all concentrations, showing a maximum of cell growth of 3% at the lowest concentration (10 µg/mL).

The high bacteriostatic effect displayed by α-amyrin phenylacetate (**4c**) against microorganisms responsible for dental caries at an initial bacteria concentration of 1 × 10^6^ cfu/mL prompted us to test its activity at a lower bacteria concentration (1 × 10^3^ cfu/mL) with the same concentrations of phenylacetate as before, and the results for *Streptococcus mutans* are shown in Figure 1. The cell viability was similar for all concentrations (around 60%), except for the 1,000 µg/mL, where the viability was 33%.

## 3. Experimental

### 3.1. General

All reagents were from Aldrich and used without further purification; chlorhexidine gluconate (CHX) was from Sigma. ^1^H-NMR and ^13^C-NMR spectra were recorded on a 400 MHz Varian instrument in CDCl_3_, with tetramethylsilane (TMS) as the internal standard. FAB mass spectra were recorded on Thermo-Electron DFS. For preparative TLC, silica Gel 60, 20 × 20 cm plates of 0.25 mm thickness (Merck) were used. The triterpenoids were detected by spraying the plates with anisaldehyde-sulphuric acid (AS) reagent, and heating at 80–100 °C for 3 min.

### 3.2. Microorganisms

The following microorganisms were used: *Streptococcus mutans* (ATCC 10449), *S. salivarius* (field strain), *S. sanguinis* (field strain), *S. oralis* (field strain), which are all associated with the oral cavity. The field strains were collected from the Medicine School of Universidad Nacional Autónoma de México (UNAM). All microorganisms were stored at −65 °C in 20% glycerol.

#### 3.2.1. Inoculum Preparation

Bacteria were refreshed in Brain Heart Infusion (BHI) broth (Difco) at 37 °C for 17 h, and later incubated at the same conditions for an additional 4 h. The optical density (OD_600_) of the *Streptococcus* culture was measured at 600 nm; an OD = 0.01 corresponds to approximately 10^6^ cells/mL.

#### 3.2.2. Statistical Analysis

The Kruskal-Wallis analysis of variance test was used for the determination of significant differences in the study [21].

### 3.3. Isolation of Triterpenes

Mexican copal resin was purchased in Tepoztlan, Morelos, and nanche bark was collected in the state of Veracruz, México. α-Amyrin (**3**) and β-amyrin (**5**) were isolated from Mexican copal resin and barks of nanche by a reported extraction procedure in 7.0% and 2.0% yield, respectively [14]. Briefly, the crude Mexican copal Tepoztlan resin (3 g) was dissolved in *n*-hexane (volume, time, temperature), and the insoluble fraction (200 mg) was used for the extraction of α-amyrin, being dissolved in dichloromethane (0.5 mL) and applied to the preparative TLC plates. After developing the plate twice in hexane-dichloromethane-methanol (10:3:0.5), the reference band was exposed to the developer (anisaldehyde-sulphuric acid), while the sample area was covered with a glass plate. Two zones (α-amyrin and a mixture of 3-*epi*-lupeol and another unidentified compound) were scraped off and each fraction was extracted three times with ethyl acetate (20 mL). The resulting solution was filtered and the solvent evaporated, yielding 160 mg (80%) of amyrin [17]. The air-dried and powdered stem barks of nance residues (1 kg) were extracted with *n*-hexane (2 L × 2) at room temperature for 48 h and 20.6 g (2.06% dry weight) of a solid white extract was obtained after filtration and evaporation of the solvent under reduced pressure. Crystallization of β-amyrin was performed by dissolving the solid white extract (4 g) in dichloromethane (20 mL) at room temperature. After dissolution, 15–20 mL of methanol was added, and the mixture was kept at room temperature for 12 h before isolation from the mother liquor by suction filtration [17].

### 3.4. Esterification of α-Amyrin

1.21 mmol of each acid **1a**–**c** were dissolved in *n*-hexane (20 mL), and oxalyl chloride (3.63 mmol) was added. The mixture was refluxed for 24 h, and then the excess of reagents was removed by evaporation under vacuum, yielding the corresponding acid chlorides **2a**–**c**, which were used without any purification. To a solution of the acid chloride in dry dichloromethane (20 mL) were added α-amyrin (0.5 mmol), pyridine (1.0 mmol) and DMAP (0.5 mmol). The resulting mixture was refluxed and monitored by TLC (Scheme 1). The reaction mixture was purified by column chromatography using different proportions of hexane-ethyl acetate, yielding the palmitate **4a**, 71%, the octanoate **4b**, 80%, and the phenylacetate **4c**, 98% isolated yields. The spectroscopic data of **4a**–**b** are consistent with those reported in the literature [18,22].

*α-Amyrin phenylacetate* (**4c**). The purified product was obtained as a white solid (98%). Rf: 0.8 hexane-ethyl acetate (90:10). IR (film) ν 3024, 2945, 2906, 1724 cm^−1^. ^1^H-NMR: δ (ppm) 0.75 (3H, s), 0.79 (3H, s), 0.90 (3H, s), 0.95 (3H, s), 1.05 (3H, s), 3.72 (2H, s, -CH_2_-Ph), 4.48 (1H, dd, *J* = 9.2, 5.6, H-3), 5.11 (1H, t, *J* = 3.6, H-12), 7.24–7.33 (5H, m). ^13^C-NMR: δ (ppm) 38.2 (C-1), 23.4 (C-2), 81.2 (C-3), 37.6 (C-4), 55.0 (C-5), 18.1 (C-6), 32.7 (C-7), 39.8 (C-8), 47.4 (C-9), 36.6 (C-10), 23.3 (C-11), 123.9 (C-12), 139.3 (C-13), 41.9 (C-14), 26.5 (C-15), 28.0 8 (C-16), 33.7 (C-17), 58.9 (C-18), 39.5 (C-19), 39.9 (C-20). MS calculated for C_38_H_57_O_2_: 545.4353; Found: 545.4336.

### 3.5. Macrodilution Broth Method for Growth Inhibition Determination

Stock solutions (1.25 and 12.5 mg/mL) of each compound **3**, **4a**–**c**, **5** were prepared in BHI broth supplemented with Tween 80 (3.6 µL/mL of broth) and sonicated before use. A series of dilutions was prepared from 10–1,000 µg/mL of α-amyrin (**3**), β-amyrin (**5**), α-amyrin phenylacetate (**4c**), α-amyrin octanoate (**4b**), and α-amyrin palmitate (**4a**). Each vial contained the *Streptococcus* strains with concentrations of 1 × 10^3^ or 1 × 10^6^ cfu/mL.

Control reactions were included consisting of inoculated growth medium without the terpene compounds; for the antimicrobial positive control, chlorhexidine gluconate (0.05%) was used. After 24 h of incubation at 37 °C, the surviving microorganisms were determined by the viable count cells.

Viable count cells: appropriate 1:10 dilutions in 0.85% sodium chloride were prepared. Each dilution (0.1 mL) was aseptically transferred to nutrient agar and the inoculum was spread with a sterile bacteriological loop. The inoculated plates were incubated for 24–48 h at 37 °C and the colonies were counted. The experiment for each dilution was done in triplicate.

## 4. Conclusions

α- and β-Amyrin were isolated from commercial crude copal resin and nanche bark, respectively. Three esters **4a**–**c** of α-amyrin (**3**) were synthesized in good to excellent yields (70–98%). To the best of our knowledge, α-amyrin phenylacetate (**4c**) is reported for the first time. Regarding biological activity, *S. mutans* was the most sensitive strain to the effect of the antimicrobial compounds evaluated in this work. α-amyrin phenylacetate showed the greatest bacteriostatic activity on an initial concentration of 10^3^ and 10^6^ cfu/mL of *S. mutans.* This property makes it a very promising compound for caries prevention.
